# The effects of professional continuous glucose monitoring as an adjuvant educational tool for improving glycemic control in patients with type 2 diabetes

**DOI:** 10.1186/s12902-021-00742-5

**Published:** 2021-04-23

**Authors:** Dulce Adelaida Rivera-Ávila, Alejandro Iván Esquivel-Lu, Carlos Rafael Salazar-Lozano, Kyla Jones, Svetlana V. Doubova

**Affiliations:** 1 Clínica de Medicina Familiar Oriente, Instituto de Seguridad y Servicios Sociales de los Trabajadores del Estado, Ciudad de México, Mexico; 2 Enseñanza e Investigación, Delegación Oriente, Instituto de Seguridad y Servicios Sociales de los Trabajadores del Estado, Ciudad de México, Mexico; 3 Delegación Oriente, Instituto de Seguridad y Servicios Sociales de los Trabajadores del Estado, Ciudad de México, Mexico; 4 Medtronic, Ciudad de México, Mexico; 5 Epidemiology and Health Services Research Unit, CMN Siglo XXI, Mexican Institute of Social Security, Av. Cuauhtemoc 330, Col. Doctores, Del. Cuauhtemoc, 06720 Mexico City, Mexico

**Keywords:** Type 2 diabetes, Professional continuous glucose monitoring, Educational tool, Glycemic control

## Abstract

**Background:**

The study objective was to evaluate the effects of professional continuous glucose monitoring (CGM) as an adjuvant educational tool for improving glycemic control in patients with type 2 diabetes (T2D).

**Methods:**

We conducted a three-month quasi-experimental study with an intervention (IGr) and control group (CGr) and ex-ante and ex-post evaluations in one family medicine clinic in Mexico City. Participants were T2D patients with HbA1c > 8% attending a comprehensive diabetes care program. In addition to the program, the IGr wore a professional CGM sensor (iPro™2) during the first 7 days of the study. Following this period, IGr participants had a medical consultation for the CGM results and treatment adjustments. Additionally, they received an educational session and personalized diet plan from a dietitian. After 3 months, the IGr again wore the CGM sensor for 1 week. The primary outcome variable was HbA1c level measured at baseline and 3 months after the CGM intervention. We analyzed the effect of the intervention on HbA1c levels by estimating the differences-in-differences treatment effect (Diff-in-Diff). Additionally, baseline and three-month CGM and dietary information were recorded for the IGr and analyzed using the Student’s paired t-test and mixed-effects generalized linear models to control for patients’ baseline characteristics.

**Results:**

Overall, 302 T2D patients participated in the study (IGr, *n* = 150; control, *n* = 152). At the end of the three-month follow-up, we observed 0.439 mean HbA1C difference between groups (*p* = 0.004), with an additional decrease in HbA1c levels in the IGr compared with the CGr (Diff-in-Diff HbA1c mean of − 0.481% points, *p* = 0.023). Moreover, compared with the baseline, the three-month CGM patterns showed a significant increase in the percentage of time in glucose range (+ 7.25; *p* = 0.011); a reduction in the percentage of time above 180 mg/dl (− 6.01; *p* = 0.045), a decrease in glycemic variability (− 3.94, *p* = 0.034); and improvements in dietary patterns, shown by a reduction in total caloric intake (− 197.66 Kcal/day; *p* = 0.0001).

**Conclusion:**

Professional CGM contributes to reducing HbA1c levels and is an adjuvant educational tool that can improve glycemic control in patients with T2D.

**Trial registration:**

ClinicalTrials.gov: NCT04667728. Registered 16/12/2020

## Background

Diabetes is a global health threat due to the high morbidity, disability-adjusted life years, premature mortality, and healthcare costs attributed to the disease. Currently, nearly half a billion people have diabetes; 90% have type 2 diabetes and 75% live in low- and middle-income countries (LMICs) [1]. In 2019, the adult population of North America and the Caribbean had the highest prevalence of diabetes globally (13.3%) and accounted for 43% of the world’s diabetes-related health expenditures [1].

Although glycemic control is the primary mechanism for preventing acute and chronic complications, disability, and premature mortality among diabetes patients [2, 3], it is achieved by only 20.9–24.9% of diabetes patients living in LMICs [4]. Laboratory testing for Hemoglobin A1c (HbA1c)—a biochemical marker of the average glycemia level over the previous 2–3 months period—is the gold standard for glucose monitoring [5]. However, HbA1c may not be an appropriate marker for patients with abnormal hemoglobin, end-stage renal disease, or chronic liver disease. Moreover, HbA1c is not an indicator for daily glucose variability, including hypoglycemic events. To assess daily blood glucose variability, determine individual glycemic targets, and provide personalized treatment of diabetes, patients must perform self-monitoring of blood glucose (SMBG). However, SMBG fails to provide a complete picture of blood glucose trends and detect hyperglycemic events [6].

Continuous glucose monitoring (CGM) with wearable devices for patients with diabetes has emerged as a method for personalizing treatment plans, aiming at improving glycemic control [7]. CGM devices utilize sensor technology inserted subcutaneously to measure interstitial glucose levels throughout the day. These devices generate glucose profile reports guiding pharmacological and non-pharmacological treatment [7]. Compared with traditional SMBG, patients with diabetes utilizing CGM achieve more significant reductions in glycated hemoglobin, body weight, and caloric intake, and higher adherence to diet and physical activity plans [8–10]. The daily glucose reports generated by CGM can help educate patients on the relationship between self-care, adherence to medication, diet, physical activity, and glycemic control [7, 11]. In addition, CGM may reduce healthcare costs through improved glucose control and decreased hospital admissions [12, 13].

There are three types of CGM tools on the market: (1) professional CGM; (2) real-time monitoring (stand-alone or connected to a pump) (RT-CGM); and (3) intermittently viewed/flash glucose monitoring (FGM). The professional CGM is a healthcare provider-managed device that generates retrospective glucose profile reports, whereas RT-CGM and FGM are managed by patients and collect real-time glucose readings [14, 15]. These sensors have demonstrated their effectiveness and there is growing evidence on their advantages, disadvantages, and indications for use [14, 15].

Mexico is a middle-income country with a high prevalence of diabetes (12%) and acute and chronic complications due to poor glycemic control [1, 16]. The Institute of Social Security and Services for State Workers (Instituto de Seguridad y Servicios Sociales de los Trabajadores del Estado—ISSSTE) provides healthcare to active and retired federal government workers and their families. In 2019, ISSSTE covered 11% (13.5 million) of Mexico’s population [17]. Currently, 1 million ISSSTE affiliates have diabetes—the second cause of death for this group [18].

Since 2007, ISSSTE has run the Comprehensive Diabetes Care program (MIDE by its Spanish acronym) to provide care to patients with HbA1c > 7% through individual consultations and self-care support groups led by multidisciplinary teams made up of family doctors, nutritionists, exercise instructors, nurses, social workers, and psychologists. Between 2007 and 2014, MIDE cared for 97,452 diabetes patients [19]. During this period, the proportion of patients with HbA1c > 7% dropped by 25%, the number of hospitalizations over a 12 month-period declined by 41, and 60% of patients reached metabolic control [19].

Although the results of MIDE are encouraging, there is still room for improvement. The present study’s objective was to evaluate the effects of professional CGM as an adjuvant educational tool for improving glycemic control in patients with type 2 diabetes.

We hypothesized that 6–7 days of professional CGM used as an adjuvant educational tool would help to decrease HbA1c levels in patients with type 2 diabetes who use this device compared with those who do not. In addition, professional CGM would help to improve patients’ dietary patterns (decrease carbohydrates, fat, protein, and total caloric intake) and CGM glucose profile (e.g., decrease time in hyperglycemic range and increase time in glucose range of 70–180 mg/dl).

## Methods

From May to October 2017, we conducted a three-month quasi-experimental study with intervention and control groups and ex-ante and ex-post evaluations in one ISSSTE family medicine clinic in Mexico City. Patients with type 2 diabetes in the MIDE program who were older than 20 years of age, had HbA1c > 8% and without diagnosis of a memory disorder were considered eligible participants for the study; those who agreed to participate, were required to sign an informed consent. From May 3 to June 15, 2017, we invited all consecutive type 2 diabetes patients who attended the MIDE program to join the intervention group; we followed the same protocol from June 16 to July 31 to assemble the control group.

The control group followed the MIDE care plan, consisting of at least two consultations with a medical doctor and HbA1c measurements at baseline and 3 months later, as well as weekly self-care educational group activities. The baseline consultation included a review of HbA1c levels and treatment adjustments.

At the beginning of the study, the intervention group had a professional CGM device (iPro™2, Medtronic, USA) inserted subcutaneously for 7 days. Before the CGM insertion, the intervention group received a training session on how to use and calibrate the device through three daily glucometer readings of capillary glucose. Moreover, intervention group patients were trained to record daily information on their medications, including the times and dosages taken; their diet, including the foods and portions consumed; and their physical activity practices. After 6 days of device use, participants had a consultation with a family physician trained in diabetes to interpret the CGM report results and adjust their treatment. In addition, a dietitian provided an educational session and personalized diet plan guided by the CGM results. Participants in the intervention group were also advised to attend regular MIDE program activities. After 3 months, the intervention group wore the sensor again for 1 week and their HbA1C levels were measured.

After the 3-month study period, the intervention and control groups continued their participation in the MIDE program.

The primary outcome variable was HbA1c level, measured at baseline and three-month evaluations in both groups. Additionally, baseline and three-month CGM data based on the 2017 international consensus on CGM metrics [20] and dietary information were recorded for the intervention group. The CGM variables included: number of days that the participant wore the CGM; percentage of time the CGM was active; mean glucose levels (mg/dl); glycemic variability measured through standard deviation (SD); time in range, defined as the percentage of time that patients’ CGM glucose readings were in the target range (70–180 mg/dl); percentage of time above 180 mg/dl and 250 mg/dl; percentage of time below 70 mg/dl and 54 mg/dl; percentage of the area over the blood concentration-time curve; and percentage of the area under the blood concentration-time curve. Dietary variables were measured through 24-h recall reports, which included measuring daily total caloric intake and caloric intake broken down by carbohydrates, proteins, and fat (Kcal/day).

The study covariates included participants’ baseline general and clinical characteristics: sex, age, educational level, nutritional status measured by body mass index (BMI: kg/m2), time since diagnosis, and pharmacological treatment (insulin, metformin, glibenclamide, pioglitazone, and linagliptin). We also recorded doctors’ modifications to treatment following the baseline HbA1c (in both groups) and CGM (in the intervention group) data.

The sample size for the primary outcome (HbA1c) was estimated using the formula to test a change in the mean of two normally distributed samples in longitudinal studies [21]. An average decrease of at least 0.7% of HbA1c in the intervention group compared with the control group was considered to be clinically relevant. Other assumptions included: α = 0.05 (for one-sided hypothesis) and a power of 90%. The number of patients by group was 143, assuming a drop-out rate of 20%.

### Statistical analysis

We performed bivariate and inferential analyses. The bivariate analysis included a comparison of the study variables between both groups. We compared the groups using the Student’s t-test for continuous variables and the Chi-square test for categorical variables. The comparison between the baseline and three-month evaluations of the CGM and dietary pattern variables in the intervention group was conducted using the Student’s paired t-test.

The intervention’s impact on the primary outcome variable (HbA1c) was assessed by estimating the differences-in-differences (Diff-in-Diff) treatment effect using a Diff-in-Diff estimator [22]. As the study lacked randomization, we adjusted the results of the Diff-in-Diff treatment effects by the participants’ baseline covariates. To control for possible missing data bias due to participant drop-out during the follow-up, we performed the study results’ sensitivity analyses using intent-to-treat analyses [23]. We conducted a Diff-in-Diff analysis for the primary outcome variable, carrying forward the baseline observation for those participants without a three-month evaluation. We did not apply the inverse probability weighting (IP-weighting) technique [24, 25] as an alternative to treat missing data given that the Diff-in-Diff analysis in STATA does not allow the use of weights.

We performed the mixed-effects generalized linear model to evaluate changes in each of the CGM and dietary variables in the intervention group and accounted for the correlation between repeated measurements (baseline and three-month). In the analysis, we controlled for patients’ baseline characteristics, such as sex, age, time since diagnosis, BMI, baseline treatment, and treatment modifications. We applied the IP-weighting technique [24, 25] to the multilevel mixed-effects generalized linear models to avoid missing data bias. This technique is based on assigning a weight to each individual with complete information, so that they account both for themselves and others with similar characteristics who have missing information; it creates a pseudo-population that eliminates missing data and where the effect of the exposure is the same as in the original population. The denominator for stabilized inverse probability weights was the probability of “having missing data” given baseline covariates, such as sex, age, BMI, time since diagnosis, and type of baseline treatment. The numerator was the probability of “having missing data” regardless of the covariates.

*P*-values < 0.05 were interpreted as statistically significant. The analyses were performed using the software Stata 14.0 (Stata Corp, College Station, TX, USA).

### Patient and public involvement

Patients or the public were not involved in the design, or conduct, or reporting, or dissemination of the present study.

### Ethics approval

The ISSSTE Ethics Committee approved the study (registry number 318.17).

## Results

Overall, 302 (87%) out of 342 invited patients agreed to participate in the study. We allocated 150 patients to the intervention and 152 to the control group. The main cited reason for not participating was a lack of time to complete the study activities.

The control (CGr) and intervention groups (IGr) had similar baseline general and clinical characteristics. Most participants were women (CGr 65.3%; IGr 71.7%); their average age ranged between 59 (IGr) and 60 years (CGr); and most had completed high school or a university degree (CGr 62%; IGr 59.2%). Both groups had a high prevalence of overweight/obesity (CGr 86%; IGr 81.6%). The average time since diagnosis was 14 years (Table 1).

**Table 1:** Participants’ characteristics

Characteristics	Control group ***n*** = 150	Intervention group ***n*** = 152
	n (%)	n (%)
**General characteristics**		
Sex, female	98 (65.3)	109 (71.7)
Age, mean (SD)	60.0 (9.2)	59.0 (9.5)
Educational level		
Elementary school or less	21 (14.0)	19 (12.5)
Secondary school	36 (24.0)	43 (28.3)
High school	62 (41.3)	60 (39.5)
University degree	31 (20.7)	30 (19.7)
**Clinical characteristics**		
Body mass index (kg/m^2^), mean (SD)	30.3 (5.7)	29.4 (4.7)
Nutritional status		
Normal weight (BMI: 18.5–24.9 kg/m^2^)	21 (14.0)	28 (18.4)
Overweight (BMI: 25.0–29.9 kg/m^2^)	63 (42.0)	61 (40.1)
Obesity (BMI ≥ 30 kg/m^2^)	66 (44.0)	63 (41.5)
Time since diabetes diagnosis (years), mean (SD)	14.3 (9.3)	14.0 (8.1)
**Baseline pharmacologic treatment**		
Insulin****	73 (48.7)	110 (72.4)
Metformin	115 (76.7)	123 (80.9)
Glibenclamide**	53 (35.3)	32 (21.1)
Pioglitazone	6 (4.0)	10 (6.6)
Linagliptin	46 (30.7)	56 (36.8)
Baseline HbA1c (%), mean (SD)***	9.3 (1.0)	9.8 (1.4)
Modifications to treatment following the baseline evaluation*	99 (66.0)	74 (48.7)
Loss of follow-up	12 (8.0)	22 (14.5)
Missing CGM information at three-month evaluation		14 (9.2%)

*SD* Standard deviation

*p < 0.05; **p < 0.01; ***p < 0.001; ****p < 0.0001

The control and intervention groups had statistically significant differences in their diabetes treatment and baseline levels of HbA1c. Compared to the control group, more IG participants had received insulin (CGr 48.7%, IGr 72.4%) and fewer had received glibenclamide (CGr 35.3%, IGr 21.1%). Additionally, the average baseline HbA1c level was higher in the intervention group (CGr 9.3%; IGr 9.8%). Following the baseline evaluation, a higher percentage of CGr participants had modifications made to their pharmacological treatment (CGr 66.0%; IGr 48.7%).

Both groups lost participants in the follow-up period; 8% of control group and 14.5% of intervention group participants did not complete the three-month evaluation. In the control group, the primary reasons for leaving the study were a lack of time or money to cover transportation costs to consultations; similarly, in the intervention group, the inability to pay for travel to the clinic for the CGM sensor insertion was cited as the main reason for discontinuing in the study. Additionally, four patients that declined the sensor’s insertion accepted the three-month HbA1C measurement. Furthermore, 14 patients who wore the sensor for the three-month evaluation had missing data in their CGM records (Table 1).

Table 2 shows the effect of the intervention on the HbA1c levels when we compared both groups. The mean HbA1C difference between the intervention and control group was 0.415% points (*p* = 0.010) for those who completed three-month evaluation and it was 0.439% points (*p* = 0.004) when the baseline observations were carried forward for 34 participants without three-month evaluations. The adjusted Diff-in-Diff estimator between intervention and control groups showed an additional decrease of HbA1c levels by 0.609 (*p* = 0.006) for patients in the intervention group who completed a three-month evaluation and an additional reduction by 0.481% points (*p* = 0.023) in the intervention group when the baseline observations were carried forward for those without three-month evaluations.

**Table 2:** Effect of the intervention on HbA1C levels

	Baseline (***n*** = 302)	Three-month (***n*** = 268)		Three-month with baseline observation carried forward (***n*** = 302)	
	Diff (T-C)	Diff (T-C)	Diff-in-Diff	Diff (T-C)	Diff-in-Diff
	β adjusted	β adjusted	β adjusted	β adjusted	β adjusted
**HbA1C**	1.024*	0.415*	− 0.609**	0.439**	−0.481*

T-intervention group; C-control group; Diff (T-C) and Diff-in-Diff estimator controlled for covariates (sex, age, educational level, nutritional status body mass index, time since diagnosis and type of pharmacological treatment) and the baseline treatment modification

*p < 0.05; **p < 0.01

Table 3 presents the results of the baseline and the three-month evaluation, and the changes in the continuous glucose monitoring and dietary patterns in the intervention group. At baseline, patients wore the CGM sensor for an average of 6.4 days, and it was active for 68.2% of the time. The mean glucose levels were 202.2 mg/dl (SD: 50.7), with low glycemic variability measured through SD 57.3 (SD: 19.0), as SD was less than the mean glucose divided by 3 [26].

**Table 3:** Continuous glucose monitoring and dietary patterns in intervention group

	Baseline evaluation		Three-months evaluation		Changes between three-months and baseline evaluation ^**a**^	
	mean (SD)	median (minimum, maximum)	mean (SD)	median (minimum, maximum)	mean	95% Conf. Interval
**Continuous glucose monitoring patterns**	***n*** **= 152**		***n*** **= 112**		***n*** **= 112**	
Number of days CGM worn	6.4 (0.61)	6 (2, 7)	6.4 (0.73)	6 (4, 8)	−0.02	− 0.21, 0.17
Percentage of time CGM was active	68.2 (18.1)	69.3 (1.1, 93.1)	72.0 (12.2)	69.2 (40.3, 98.9)	3.03	−0.74, 6.81
Mean glucose levels, mg/dl	202.2 (50.7)	198 (103, 397)	190.9 (51.9)	181.5 (104, 362)	−11.64*	−22.92, −0.36
Glycemic variability measured through standard deviation	57.3 (19.0)	56 (11, 115)	53.3 (17.8)	52.5 (13, 111)	−3.93*	−7.57, − 0.29
Percentage of time in range (70–180 mg/dl)	43.4 (25.9)	40 (0, 93)	50.9 (28.7)	52.5 (0, 100)	7.65*	1.86, 13.44
Percentage of time above range (> 180 mg/dl)	54.9 (27.0)	58 (1, 100)	48.3 (29.4)	46 (0, 100)	−6.57*	−12.73, −0.41
Percentage of time above range (> 250 mg dl)	23.9 (23.2)	17.5 (0, 100)	20.3 (23.3)	11.5 (0, 96)	−3.0	−8.0, 1.9
Percentage of time below range (< 70 mg/dl)	1.4 (3.5)	0 (0, 21)	0.9 (2.2)	0 (0, 14)	−0.49	−1.26, 0.27
Percentage of time below range (< 54 mg/dl)	0.9 (4.9)	0 (0, 51)	0.2 (0.8)	0 (0, 5)	−0.86	−1.91, 0.19
Percentage of the area over the blood concentration-time curve	61.3 (43.1)	52 (0.3, 246.9)	52.7 (43.6)	43 (0, 212.3)	−8.50	−17.93, 0.93
Percentage of the area under the blood concentration-time curve	0.22 (0.78)	0 (0, 6.4)	0.09 (0.29)	0 (0, 1.6)	−0.13	−0.29, 0.04
Dietary patterns						
Daily caloric intake, Kcal/day	***n*** **= 152**		***n*** **= 122**		***n*** **= 122**	
Total	1334.6 (443.7)	1250 (538, 3157)	1144.8 (295.6)	1108.5 (456, 2085)	−189.83*	−270.6, −109.0
From carbohydrates	603.7 (235.3)	576 (68, 1184)	507.9 (200.7)	500 (72, 892)	−95.80*	− 141.62, −49.99
From proteins	288.3 (92.9)	272 (132, 652)	255.7 (76.7)	248 (108, 420)	−32.66*	−49.77, −15.55
From fat	431.9 (235.1)	387 (0, 1620)	375.9 (124.2)	378 (0, 747)	−56.06*	− 101.58, − 10.55

*SD* Standard deviation

*p < 0.05

The CGM glucose readings reported a time in range of 43.4% (the percentage of time that patients were in the target glucose range) (SD: 25.9), 54.9% (SD: 27.0) above 180 mg/dl, and 1.4% (SD: 3.5) below 70 mg/dl. The percentage of the area over the blood concentration-time curve was 61.3 (SD: 43.1), and the percentage of the area under the blood concentration-time curve was 0.22 (SD: 0.78).

Regarding caloric consumption, patients in the intervention group consumed 1334.6 Kcal/day (SD: 443.7); an average of 603.7 Kcal/day (SD: 235.3) were from carbohydrates and an average of 431.9 Kcal/day (SD: 235.1) were from fat.

Compared with the baseline, the three-month CGM patterns showed a significant improvement in patients’ metabolic control. We observed a reduction in the mean glucose levels (− 11.64; 95% confidence interval [95% CI]: − 22.92, − 0.36, *p* = 0.0216) and glycemic variability (− 3.93; 95% CI: − 7.57, − 0.29); there was also a substantial increase in the percentage of time in range (mean increase of + 7.65; 95% CI:1.86, 13.44, *p* = 0.0050) and a reduction in the percentage of time above 180 mg/dl (mean reduction of − 6.57; 95% CI: − 12.73, − 0.41, *p* = 0.0184). Moreover, we noted a decrease in total caloric intake (− 189.83 Kcal/day; 95% CI: − 270.6, − 109.0, *p* = 0.00001), including a reduction in calories from carbohydrates (−95.8 Kcal/day; 95% CI: − 141.62, − 49.99, p = 0.00001), followed by fat (− 56.06 Kcal/day; 95% CI: 101.58, − 10.55, *p* = 0.0081), and protein (− 32.66 Kcal/day; 95% CI: − 49.77, − 15.55, *p* = 0.0001).

Table 4 depicts that the positive effects of CGM remained statistically significant after controlling for patients’ baseline characteristics and missing data in the intervention group. The effects include: a decrease in glycemic variability (− 3.94; 95% CI: − 7.59, − 0.29, *p* = 0.034), an increase in the percentage of time in range (+ 7.25; 95% CI: 1.65, 12.85, *p* = 0.011); a reduction in the percentage of time above 180 mg/dl (− 6.01; 95% CI: − 12.08, − 0.13, *p* = 0.045); and improvements in dietary patterns, shown by a reduction in calories from carbohydrates (− 95.68 Kcal; 95% CI: − 141.95, − 49.42, *p* = 0.0001), fat (− 62.75 Kcal; 95% CI: − 112.83, − 12.67, *p* = 0.014), and protein (− 33.61 Kcal/day; 95% CI: − 50.68, − 16.54, p = 0.0001) and a reduction in total caloric intake (− 197.66 Kcal/day; 95% CI: − 282.87, − 112.467, *p* = 0.0001).

**Table 4:** Changes in continuous glucose monitoring data and dietary patterns in intervention group after controlling for patients’ baseline characteristics and missing data

	IP-weighted Coefficient	IP-weighted 95% Confidence Intervals
Mean glucose levels, mg/dl	−10.44	−21.83, 0.96
Glycemic variability measured through standard deviation	−3.94*	−7.59, − 0.29
Percentage of time in range (70–180 mg/dl)	7.25*	1.65, 12.85
Percentage of time above range (> 180 mg /dl)	− 6.10*	− 12.08, − 0.13
Percentage of time above range (> 250 mg /dl)	−3.05	− 8.03, 1.93
Percentage of time below range (< 70 mg/dl)	− 0.63	−1.39, 0.12
Percentage of time below range (< 54 mg/dl)	− 0.93	− 1.97, 0.09
Percentage of the area over the blood concentration-time curve	−8.01	− 17.5, 1.50
Percentage of the area under the blood concentration-time curve	− 0.15	− 0.32, 0.01
Dietary patterns		
Daily caloric intake, Kcal/day		
Total	− 197.66***	− 282.87, − 112.467
From carbohydrates	− 95.68***	−141.95, − 49.42
From proteins	−33.61***	−50.68, − 16.54
From fat	−62.75*	− 112.83, − 12.67

Each model was controlled for covariates (sex, age, educational level, nutritional status body mass index, time since diagnosis and type of pharmacological treatment) and the baseline treatment modification

*p < 0.05; **p < 0.01; ***p < 0.001

## Discussion

This study showed significant positive effects of professional CGM as an adjuvant educational tool for improving glycemic control of patients with type 2 diabetes, as reflected in the additional 0.481% points decrease of HbA1c levels in the intervention group compared with the control group in the three-month follow-up. The intervention group reached a significant increase in the time in range measurement and decreases in the time above 180 mg/dl, glucose variability, and total caloric intake.

In our study, physicians and dietitians used CGM information to adjust pharmacological and dietary treatment and to educate patients on self-care based on detailed and patient-friendly glucose pattern summaries and glucose profile curves. Previous studies have shown the potential of professional CGM as an educational tool for improving glycemic control in patients with type 1 and type 2 diabetes [7, 9]. Professional CGM generates a simplified report that has standardized statistics, targets, and visual representations of glucose patterns over time, which can educate patients on how insulin, daily activities, and diet impact their blood glucose levels. This information can inform and empower patients in their treatment decision-making, as well as their understanding of treatment plans and patient-centered treatment goals [7–10, 27].

Routine blood glucose measurement methods show disadvantages when compared to CGM. For example, HbA1c measurements and patient self-monitoring of blood glucose do not identify glucose variability. Daily self-monitoring of blood glucose is a static snapshot of glucose levels, and it is painful and challenging to maintain [28]; its use ranges between 2.7 and 4.4 times/day [29]. Meanwhile, professional CGM provides continuous information on glycemic control beyond HbA1c, thus identifying glucose variability and timing related to hyperglycemia and hypoglycemia [7]. Although professional CGM is a retrospective method, the healthcare practice in general is moving towards real-time monitoring that generates data that is accessible to patients, and which can be remotely shared with health professionals and used to develop an artificial pancreas system [30].

Our intervention improved glucose parameters and caloric intake; namely, a significant increase in the time in range and decrease in hyperglycemia, glucose variability, and caloric intake in the intervention group. These results align with similar interventions that have shown a decrease in glucose variability and caloric intake in patients with type 2 diabetes after using CGM to guide their pharmacological and non-pharmacological treatment [7, 9]. For instance, a study from South Korea showed that CGM monitoring in patients with type 2 diabetes for 3 days monthly over 3 months modified patients’ glucose profile and reduced their caloric intake by 168.7 kcal/day [31]. Another three-month observational study from the United States showed that a lifestyle intervention combined with CGM reduced total energy intake by 884 Kcal and total carbohydrate intake by 92.8 g [32].

The mean reduction of HbA1c levels by 0.481% points 3 months after its application in the intervention group compared with the control group demonstrates additional value of CGM. This result is consistent with previous studies. A recent CGM systematic review that included seven randomized controlled trials and three cohort studies involving 1384 patients with type 2 diabetes with RT-CGM and P-CGM and 4902 patients with FGM, showed that CGM was associated with a modest but statistically significant reduction in HbA1c of 0.20% (95% CI − 0.31 to − 0.09) compared with traditional glucose self-monitoring [8]. This finding is noteworthy as HbA1c is a reliable biomarker for diabetes diagnoses and measures chronic hyperglycemia, which is correlated with long-term diabetes complications [33]; it is also associated with an increased risk for cardiovascular complications and all-cause mortality among patients with type 2 diabetes [34]. The relative risk associated with a 1% increase in HbA1c level among patients with type 2 diabetes was 1.15 (95% CI, 1.11 to 1.20) for all-cause mortality and 1.17 (95% CI, 1.12 to 1.23) for cardiovascular disease [34]. Furthermore, it is reasonable to assume that the use of CGM and consequent improvements in HbA1C levels could reduce the use of healthcare services and the corresponding costs of care. This assumption is supported by several studies that have compared healthcare utilization and costs of patients with and without real-time CGM [12, 13, 35].

The study has several limitations. First, it was a pilot study with intervention and control groups and ex-ante and ex-post evaluations in one family medicine clinic. This design is “quasi-experimental” because assignment into the intervention and control groups was not random, which could compromise the interpretation of results if baseline measurements differed between the groups, as they did for baseline treatment and HbA1c levels in our study. We addressed these dissimilarities by using the Diff-in-Diff estimator analysis. Second, we did not document study participants’ adherence to pharmacological and non-pharmacological recommendations (e.g., regular physical exercise). Third, the dietary and glucose patterns were only measured in the intervention group due to logistical restrictions. Fourth, the dietary patterns were assessed through self-reporting, which risks over- or under-estimating the effect of the intervention. Nonetheless, most studies on primary care services have applied this type of questionnaire because of its feasibility.

## Conclusion

Primary care services deliver a majority of care for patients with type 2 diabetes. Care for these patients is complex and requires interprofessional collaboration. Our results indicate that in the context of Latin American countries, the professional CGM may be a useful tool for accurate data that can guide the therapeutic and educational decisions of health providers treating patients with type 2 diabetes. Further studies to ascertain the feasibility, cost-effectiveness, and other potential benefits of expanding the use of this technology in healthcare settings could further inform decision-making.

## Data Availability

The raw database generated and analysed during the current study are not publicly available, as this was not part of the participant consent process; however, the database is available from the corresponding author on reasonable request.

## Abbreviations

BMI: Body mass index
CGM: Continuous glucose monitoring
95%CI: 95% confidence interval
CGr: Control group
Diff-in-Diff: Differences-in-differences
FGM: Intermittently viewed/flash glucose monitoring
HbA1c: Hemoglobin A1c
IGr: Intervention groups
IP-weighting: Inverse probability weighting
ISSSTE: Institute of Social Security and Services for State Workers (Instituto de Seguridad y Servicios Sociales de los Trabajadores del Estado)
LMICs: Low- and middle-income countries
MIDE: Comprehensive Diabetes Care program (MIDE by its Spanish acronym)
RT-CGM: Real-time monitoring (stand-alone or connected to a pump)
SMBG: Self-monitoring of blood glucose
SD: Standard deviation
